# Anxiety and depression among pregnant women undergoing IVF-ET and WeChat group peer support during the COVID-19 pandemic: Study protocol for a randomized controlled trial

**DOI:** 10.1097/MD.0000000000032515

**Published:** 2022-12-23

**Authors:** Jing-Xian Cao, Wen-Jing Jiang, Meng-Han Yan, Dan-Dan Wang, Jin-Wei Hou, Jing-Yan Song, Zhen-Gao Sun

**Affiliations:** a The First Clinical College, Shandong University of Traditional Chinese Medicine, Jinan, China; b College of Traditional Chinese Medicine, Shandong University of Traditional Chinese Medicine, Jinan, China; c Reproductive Center of Integrated Medicine, The Affiliated Hospital of Shandong University of Traditional Chinese Medicine, Jinan, China.

**Keywords:** anxiety, depression, peer support, wechat, in vitro fertilization and embryo transfer

## Abstract

The psychological well-being of pregnant women following assisted reproductive has increasingly gained attention in recent years. Anxiety and depression may be associated to pregnancy outcomes. This study aims to determine whether peer support and the WeChat group platform will reduce anxiety and depression among in vitro fertilization and embryo transfer (IVF-ET) women.

**Methods and Analysis:**

In the present randomized controlled study, 296 patients with confirmed clinical pregnancy following IVF-ET will be randomly assigned to receive standard intervention support or WeChat peer support on a 1:1 basis. The levels of anxiety and depression are the primary endpoints. Assessments will be performed at baseline measurements, first trimester, second trimester, and third trimester, and data will be collected.

**Ethics and Dissemination:**

This study has been approved as ethical by the affiliated hospital of Shandong University of Traditional Chinese Medicine’s Reproductive Ethics Committee. Each patient will sign a written statement of informed permission. All information and biological samples will be legally protected. A peer-reviewed academic journal will publish the findings of this investigation.

**Discussion:**

Given the inconvenience of visits due to the current pandemic of COVID-19, this study addresses the patient’s visit needs by combining WeChat, the most widely used social software in China, with peer support, while helping improve maternal anxiety, depression, and pregnancy outcomes following IVF-ET.

## 1. Introduction

The COVID-19 outbreak has had a substantial impact on patient medical treatment procedures as well as emotional and mental health states. Home isolation decreases patients’ compliance with medical care and medicines, and it also raises some residents’ anxiety, despair, sleep problems, and other negative emotions, according to a poll.^[1,2]^ Psycho-emotional disorders in infertility patients have gotten more attention as the medical paradigm evolves from biomedical to bio-psycho-social,^[3–5]^ and these unpleasant emotions may cause varied degrees of melancholy, anxiety, pain, and a deterioration in life quality.^[6]^ Numerous studies have shown that advanced reproductive technology-seeking couples are more likely than naturally conceived couples to endure psychological anguish.^[7]^ The consumption of large amounts of time and money, the uncertainty surrounding the outcome of treatment, and the worries surrounding the course of treatment may all contribute to anxiety and depression. Additionally, anxiety over early parenting challenges, fetal survival, and pregnancy outcomes is also higher.^[8–12]^ These psychological aspects are crucial to in vitro fertilization and embryo transfer (IVF-ET) treatment and may even have an impact on pregnancy outcomes. Women undergoing IVF-ET may experience difficulty getting pregnant, and the psychological stress that follows pregnancy remains. Unfavorable feelings will adversely affect the development of the fetus and the maternal and fetal endocrine systems.^[13]^ The fetal nervous system’s development and the fetus’s functioning of the hypothalamic-pituitary-adrenal (HPA) axis have both been found to be negatively impacted by maternal anxiety, depression, and other negative emotions.^[14,15]^ As preterm deliveries, miscarriages, fetal intellectual disabilities, and congenital malformations are significantly higher in pregnant women, anxiety and depression are independent risk factors for poor pregnancy outcomes.^[16,17]^

It’s not a new concept to use peer support to recognize and treat various health concerns. A peer support program is a method used to enable people with similar illnesses, bodily problems, or experiences to help 1 another, as well as to provide social, emotional, and practical support.^[18]^ People with similar experiences are more able to bond, which can lead to more genuine empathy and validation, according to Mead and MacNeil’s “generic” definition.^[19]^ Self-regulation appears to be non-coercive, reciprocal, and decentralized and can be supported by social norms, identity, and friendship.^[20]^ Peer support is acknowledged for having a significant positive influence on underrepresented and difficult-to-reach groups and plays a role in humanizing healthcare.^[21]^ Self-management of chronic diseases such as cancer, diabetes, hypertension, etc, has been proven to improve patients’ physical, psychological, and health outcomes.^[22–24]^ There have been numerous studies regarding breastfeeding, all of which have shown positive results.^[25]^ A number of studies have demonstrated that, although a lack of social support is associated with depression and anxiety prenatally and postnatally, 1’s sense of other people’s availability as resources significantly increases 1’s capacity to regulate 1’s own distress.^[26–28]^ Several interrelated benefits of peer support have been identified as enhancing the emotional wellbeing of mothers during pregnancy and postpartum,^[21]^ as of yet, no studies have been conducted on pregnancies achieved through assisted reproduction.

In recent years, the Internet’s rapid development and the evolution of information technology have changed how medicine is practiced. A new word, e-health, is used to describe health services and information that are supplied or improved via electronic procedures and communications.^[29]^ In nations with limited medical resources, like China, high smartphone penetration and extensive adoption of social media applications present hitherto unheard-of prospects for mobile phone-based e-health interventions. Several studies have demonstrated the effectiveness of smartphones and social media in promoting health habits, which suggests that booming e-health can significantly impact chronic disease treatment.^[30,31]^ All in all, electronic medical care has been extremely important during the outbreak.

Mobile health apps are revolutionizing how doctors consult with patients in China. WeChat has grown to be the most popular and widely used social media platform in China and is an essential component of Chinese citizens’ daily lives.^[32]^ It is simple to use and offers group chats, text and voice messaging, free audio and video calls, access to official accounts and mini-programs, etc. WeChat makes getting health information more convenient, quick, and need-based. Numerous hospitals have implemented programs for health counseling, health evaluation, health education, and health promotion centered on helping patients with chronic illnesses live healthy lifestyles and eat sensibly while also fostering positive doctor-patient relationships. WeChat groups have been used to share disease-related information with patients, which has increased their medication compliance.^[33,34]^ Patients with chronic diseases can benefit from continuous, prompt, and thorough follow-up interventions in order to achieve the goals of early detection, early treatment, and early prevention. Data collected by WeChat can be efficiently gathered, a sizable database can be created, and data support can be provided to enhance diagnosis and treatment. In conclusion, WeChat-based health interventions are less expensive, more effective at getting patients to comply with their treatment plans, less likely to cause complications, and have higher follow-up rates. Thus, traditional medical resources are balanced, effective physician-patient communication is encouraged, and public resources are reduced.^[35–37]^

In contrast, research on WeChat group applications for pregnant women and assisted reproduction is scarce. Therefore, the aim of this study is to establish a WeChat group that provides peer support to pregnant women undergoing IVF-ET to assist them in coping with anxiety and depression during pregnancy. Consequently, it is expected to serve as a resource for clinical decision-making.

## 2. Methods and analysis

### 2.1. Study design

**Figure 1.**
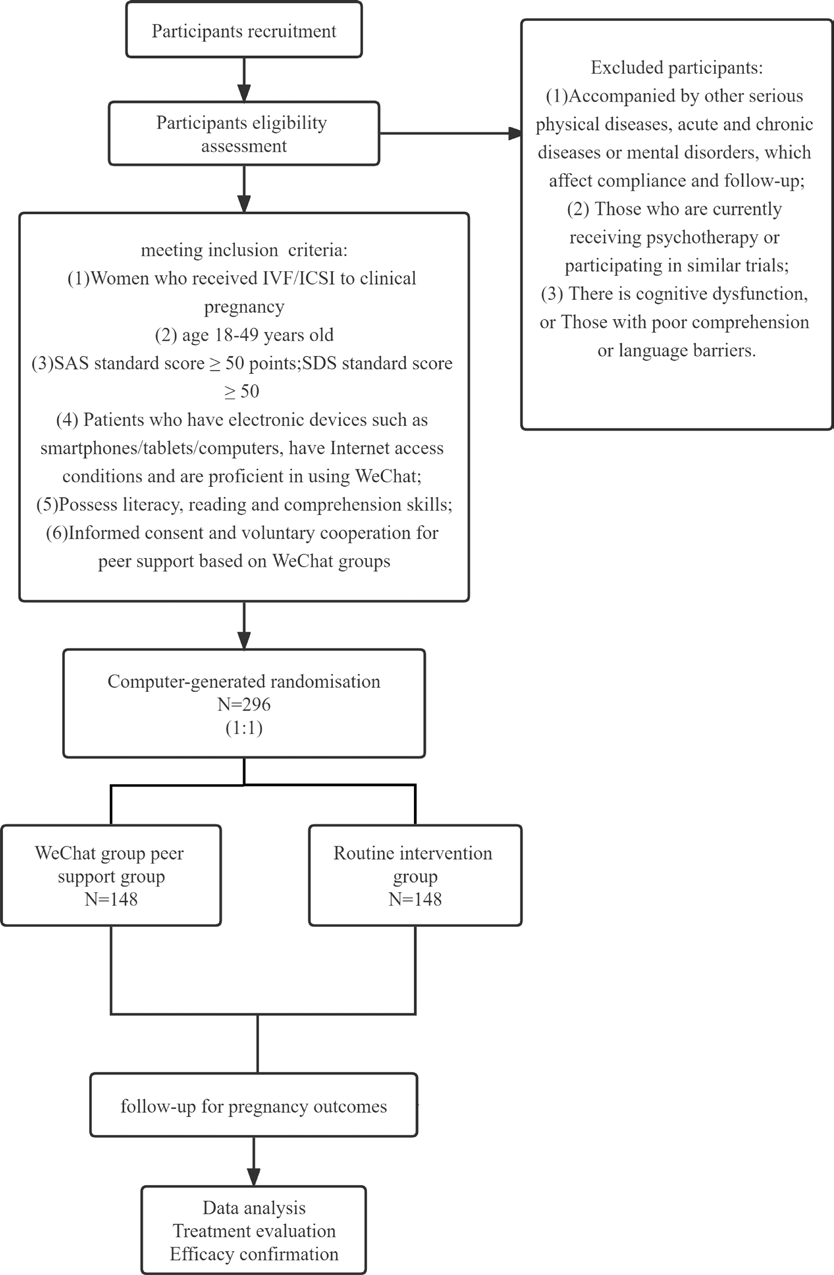
The study flow chart.

A randomized control trial design will be used in this study. It is anticipated that recruitment at the Reproductive Center of Integrated Medicine, Affiliated Hospital of Shandong University of Traditional Chinese Medicine (SDUTCM) will begin in December 2022 and last through December 2023 for women with confirmed clinical pregnancy undergoing IVF-ET. Writing this guideline, we adhered to SPIRIT’s suggestions.^[38]^ Figure 1 displays the research flow chart, and Table 1 displays the research process timetable.

**Table 1:** Example template of recommended content for the schedule of enrolment, interventions, and assessments. *

Outcome	Assessment	Baseline	11–13 wks gestation	19–21 wks gestation	27–29 wks gestation	Within 3 d after delivery
**Primary outcome**						
Anxiety level	SAS core	√	√	√	√	√
Depression level	SDS core	√	√	√	√	√
**Secondary outcome**						
pregnancy outcome						
ectopic pregnancy			√	√	√	
miscarriage			√	√	√	
early pregnancy loss			√			
late pregnancy loss			√	√	√	
premature birth						√
live birth						√

SAS = self-rating anxiety scale, SDS = self-rating depression scale.

### 2.2. Data collection

Both groups of patients are required to provide baseline information, including sociodemographic characteristics, disease history, and social behavioral characteristics such as smoking and alcohol use. Collect and complete the self-rating anxiety scale (SAS), which consists of 20 items overall, of which 5 indicate positive emotional experience and 15 negative emotional experience; the greater the score, the more anxious the patient is; the score below 50 represents normal, while mild will be anxiety score of 50 to 59, moderate anxiety 60 to 69, severe anxiety 70 points or more, and self-rating depression scale (SDS) (a total of 20 items; the greater the score, the higher the level of anxiety). Less than 50 points are considered normal, 50 to 59 points are considered mild anxiety, 60 to 69 points are considered moderate anxiety, and more than 70 points are considered severe anxiety during the course of treatment.

### 2.3. Eligibility criteria

#### 2.3.1. Inclusion criteria.

1. Clinically pregnant women (i.e., fetal heartbeat or gestational sac at 6–9 weeks after embryo transfer^[39]^) undergoing IVF-ET.
2. age 18 to 49 years old.
3. SAS standard score ≥ 50 points (i.e., mild anxiety or above); SDS standard score ≥ 50 points (i.e., mild depression or above).
4. Patients who own electronic devices such smartphones, tablets, or computers, have Internet access, and are skilled WeChat users.
5. Have reading, writing, and comprehension abilities.
6. Informed consent and willing participation for WeChat group-based peer support.

#### 2.3.2. Exclusion criteria.

1. Accompanied by other serious physical diseases, acute and chronic diseases or mental disorders, which affect compliance and follow-up.
2. Those who are undergoing psychotherapy at the moment or taking part in comparable studies.
3. There is cognitive dysfunction, or those with poor comprehension or language barriers.

#### 2.3.3. Dropout criteria.

After recruitment, participants who failed to comply with the clinical study protocol, did not match the inclusion criteria, did not follow conventional treatment practices, or refused to give their permission should be removed. Participants who fulfilled the eligibility requirements but withdrew from the clinical trial for a variety of causes, such as individuals who voluntarily withdrew or when the researchers became aware that the subjects had withdrawn.

### 2.4. Study case discontinuation

Subjects will be removed from the study if they exhibit unusual physiological reactions, experience other unanticipated events, or are deemed unfit to continue taking part in the experiment. In the event that the individual experiences significant issues or if their health worsens, immediate action will be done.

### 2.5. Study population and recruitment

Patients having clinical pregnancies following IVF-ET will start enrolling in the trial. Once clinical pregnancy is confirmed and the inclusion and exclusion requirements are met, patients will receive comprehensive written information regarding the trial methodology and give their signed informed permission. Baseline blood tests, ultrasonography, anxiety and depression rating scales, as well as basic demographic data, will all be completed. After receiving the trial protocol, the researchers will allocate patients to one of the study groups randomly. All participants in the study are free to leave at any moment without providing a reason.

### 2.6. Interventions

Peer support group on WeChat:

1. Ten women who had received IVF-ET in our center and had given birth at term were chosen as peer support volunteers in the WeChat group. These ladies had good return visit records. Selection criteria include: (1) a happy disposition, the ability to communicate oneself clearly and persuasively, (2) a sound mental condition, as well as strong self-control and self-discipline, (3) completion of primary school or higher, as well as adequate leisure.
2. Ten volunteers underwent training from attending doctors and specialized nurses that covered common illnesses after IVF-ET during pregnancy, interactive skills for using relevant WeChat groups, job responsibilities and team management skills, and knowledge of how to deal with typical psychological issues. Five lessons altogether, lasting between 1 and 2 hours each. Individualized online or offline tuition can be offered depending on the needs of the team leader.
3. A WeChat group called “IVF-ET Peer Support Exchange Group” was created. Its members included 148 subjects, 10 volunteers, an attending physician, and a specialized nurse. According to the participants’ data, volunteers routinely reminded them to have their obstetric exams, provided weekly pregnancy-related information on WeChat’s public account, and disseminated pregnancy advice. Weekly group video/group audio communications offered the subjects emotional assistance. Daily prenatal education music was shared with the participants as well as reminders to lead healthy lives and get enough sleep (this included a nutritious diet, cutting back on late nights, quitting smoking and drinking, and having a happy outlook). At any point during the course of treatment, patients are free to ask questions and express their feelings in the group, and volunteers are there to clear up any confusion. Attending physicians and specialty doctors can offer focused explanations and emotional support when patients feel disputed or bewildered.

### 2.7. Routine intervention group

Regular obstetric exams, departmental pregnancy awareness booklets, routine health education about emotions, diet, sleep, and other pregnancy-related topics, as well as routine health education will be given to the subjects.

At 11 to 13 weeks in the first trimester, 19 to 21 weeks in the second trimester, and 27 to 29 weeks in the third trimester, the individuals in both groups will complete the electronic questionnaire survey that asked about their levels of anxiety and sadness.

### 2.8. Outcomes

After participant enrollment, baseline evaluations will be done, then participants will have follow-up appointments at 11 to 13 weeks in the first trimester, 19 to 21 weeks in the second trimester, and 27 to 29 weeks in the third trimester. Additionally, 2 groups of people will receive electronic questionnaires for anxiety and depression levels to gauge the effectiveness of the intervention. Primary endpoint measures are improvements in anxiety and depression (i.e., overall effectiveness rate, defined as the proportion of patients with a decreases of more than 25% in SAS^[40]^ and SDS^[41]^ scores following the abovementioned intervention). At each follow-up, the anxiety and depression ratings of the 2 groups are compared horizontally, and the simultaneous effects of the 2 interventions on the subjects’ anxiety and depression are compared. The anxiety and depression ratings of the 2 groups are longitudinally compared after all the follow-up have been completed, and the effects of the 2 interventions on the participants’ anxiety and depression before and after the interventions are compared. The secondary outcomes are comparisons of pregnancy outcomes, including ectopic pregnancy, miscarriage, as well as live birth. Ectopic pregnancy is defined as the development of the fertilized egg outside the uterine cavity; miscarriage is defined as termination of pregnancy at less than 28 weeks of gestation with a fetus weighing less than 1000 g; live birth is defined as at least 22 weeks of gestation or at least 500 g.

### 2.9. Randomization and blinding

After informed consent, subjects will be assigned 1:1 by computer to one of the WeChat group peer support group or the routine intervention group using a random code sequence in the order of registration. Randomization protocols are entered into the online central randomization database (www.medresman.org). Given the apparent difference in intervention strategies between the 2 groups, neither investigators nor participants were blinded; however, trial outcome assessors will be blinded.

### 2.10. Data management, informed consent and confidentiality

Data will be electronically recorded. Electronic copies will be kept on a password-protected computer in encrypted files. For 15 years, all data will be retained. The digital information will then be prepared for deletion. The questionnaires will be kept in the center’s repository for experimental research and will only be accessible to authorized staff during the course of the study. There is no personally identifying information in the participant records or surveys.

### 2.11. Sample size calculation

Overall effectiveness rate is defined as the proportion of patients whose SAS and SDS scores have reduced by more than 25%, the primary endpoint of the study. Based on previous clinical experience in our center, at the third follow-up visit, we found that the overall effectiveness rate was approximately 75% in the WeChat peer support group and about 60% in the routine intervention group. A total of 236 patients were enrolled in the study utilizing the PASS 15 procedure (NCSS, LLC. Kaysville, Utah), with a hypothesis test level of 0.05 and a test power of 0.8. After accounting for the 20% dropout rate, 296 patients with 148 in each group comprised the final sample size.

### 2.12. Statistical analysis

The SPSS 26.0 software was used to analyze all the data (SPSS Inc., Chicago, IL). The data’s normality was evaluated using the Shapiro–Wilk test. Depending on the distribution’s normality, quantitative variables were examined using the independent samples t-test or Mann–Whitney *U* test and represented as mean standard deviation (SD) or median (interquartile range, IQR). Frequencies and percentages are used to express qualitative characteristics. Comparing categorical variables was done using the 2 test or Fisher’s exact test. In all statistics, a 2-tailed alpha of 0.05 was employed.

### 2.13. Quality control and assurance

The subjects were selected in strict accordance with the protocol, and the communication with the subjects was strengthened to ensure follow-up and compliance. Each subject establishes an electronic follow-up record card to record the implementation of the intervention plan, the visit plan, the precautions during the study, the report of emergency events, etc, according to the established follow-up time and the implementation of the intervention measures. And improve the operation specifications of various instruments, and conduct regular quality control inspections on major equipment.

### 2.14. Patient and public engagement

The study comprised women who underwent IVF-ET at the Reproductive Center of Integrated Traditional Chinese and Western Medicine, Affiliated Hospital of SDUTCM, and had a confirmed clinical pregnancy as a result. No patients or members of the public were involved in the development of the study topic, study design, study conduct, interpretation of the study results, or editing and publication of the final manuscript. Their doctors will be able to communicate the results with the participants.

## 3. Discussion

Anxiety and depression are common in women who experience clinical pregnancy undergoing IVF-ET, which has a significant negative impact on both quality of life and pregnancy outcomes. How people seek care is affected by COVID-19 quarantines. As a result of poor occupancy rates per capita and unequal distribution of medical resources, traditional patient follow-up approaches are currently insufficient to handle the needs of a large number of patients.^[42]^ This study’s design is built on the WeChat group platform, which gives subjects peer support. Previous studies have shown that the WeChat group peer support can combine encouragement, support, and health education to effectively teach patients to strengthen self-management and improve living habits, effectively control overall medical expenses.^[31]^

In our study, researchers and volunteers regularly send health education texts, pictures, videos and other information through group chat or video or voice, which can break the mobile phone short message service affected by factors such as high cost, limited management, and boring text^[43,44]^ and telephone interviews^[45,46]^ and other traditional modes of information exchange. Pregnant women can interact effectively in a group conversation on WeChat. Peer support volunteers who have had children themselves can offer individualized dynamic communication to help subjects through direction and communication whenever and wherever they need it. Additionally, students can discover practical techniques and strategies for enhancing self-management, establishing a healthy lifestyle, and fostering positive outcomes. Anxiety and depression scores were calculated for 2 groups of subjects in the first, second, and third trimesters of pregnancy in conjunction with a peer support intervention in a WeChat group. Data was gathered using questionnaires, which were easy to use, cost-effective, and favorable to data collection, storage, and administration. In the future, we can work with professionals to create specialized applets that can be used throughout the assisted reproductive therapy process, giving patients affordable, practical, and efficient options.

In conclusion, we design this randomized controlled study to examine whether an intervention based on WeChat group peer support can improve anxiety, depression, and pregnancy outcomes in pregnant women following IVF-ET. This is done to ensure the medical conditions of patients under special circumstances.

## Author contributions

**Data curation:** Jing-Xian Cao, Jing-Yan Song, Wen-Jing Jiang, Meng-Han Yan, Dan-Dan Wang, Jin-Wei Hou.

**Resources:** Zhen-Gao Sun.

**Writing – original draft:** Jing-Xian Cao.

**Writing – review & editing:** Jing-Yan Song, Zhen-Gao Sun.
